# Thyroid Storm in a Patient With COVID-19

**DOI:** 10.1016/j.aace.2021.06.011

**Published:** 2021-07-03

**Authors:** Angela N. Rao, Ruaa Y. Al-Ward, Ruchi Gaba

**Affiliations:** 1 Department of Internal Medicine, Baylor College of Medicine, Houston, Texas; 2 Department of Internal Medicine, Section of Endocrinology, Baylor College of Medicine, Houston, Texas

**Keywords:** COVID-19, SARS-CoV-2, thyroid, thyroid storm, NR, normal range, T3, triiodothyronine, T4, thyroxine

## Abstract

**Objective:**

A thyroid storm is a severe exacerbation of thyrotoxicosis that can cause significant morbidity and mortality. The emergence of the novel coronavirus (SARS-CoV-2) that originated in Wuhan, China, has become a worldwide pandemic. We present the first documented case of thyroid storm (as defined by the Burch-Wartofsky criteria) in a patient with COVID-19.

**Methods:**

Laboratory and diagnostic studies, including thyroid function tests, thyroid antibody testing, SARS-CoV-2 nasopharyngeal polymerase chain reaction testing, and thyroid ultrasound were performed.

**Results:**

A 25-year-old woman presented to the hospital with dry cough, dyspnea, palpitations, weight loss, diarrhea, and anxiety. Physical examination revealed exophthalmos with proptosis and chemosis, tachycardia, diffusely enlarged goiter with bruit, and fine tremor. Laboratory results demonstrated a thyroid-stimulating hormone level of <0.01 mIU/L (normal range [NR], 0.44-5.3 mIU/L), free thyroxine level of 5.34 ng/dL (NR, 0.64-1.42 ng/dL), total triiodothyronine level of 654 ng/dL (NR, 87-178 ng/dL), and thyroid-stimulating immunoglobulin level of 7.18 IU/L (NR, 0.00-0.55 IU/L). Thyroid ultrasound revealed heterogeneous echotexture with increased vascularity. Nasopharyngeal COVID-19 testing was positive. She was treated promptly with propranolol, propylthiouracil, and hydrocortisone with improvement in symptoms, and later switched to methimazole. Her COVID-19 course was uncomplicated, and she left the hospital with minimal respiratory symptoms.

**Conclusion:**

Thyroid storms are one of the more prevalent endocrine emergencies and are often precipitated by acute events including infections. Patients with thyroid storms may have concomitant SARS-CoV-2 infection that could influence the clinical course and severity of the disease. In patients with symptoms of thyrotoxicosis and respiratory symptoms, clinicians should consider performing a COVID-19 test.

## Introduction

A thyroid storm is defined as a life-threatening exacerbation of thyrotoxicosis, and is characterized by tachycardia, hyperthermia, agitation, altered mental status, and other organ system dysfunctions. It is a clinical emergency that, without an early diagnosis and treatment, can be fatal, with recent sources reporting a mortality rate anywhere from 3.3% to 25%.^1^, ^2^, ^3^ Since January 2020, COVID-19 caused by SARS-CoV-2 infection has been known to precipitate endocrinologic emergencies such as diabetic ketoacidosis, and can result in thyroid-related complications including subacute thyroiditis.^4^^,^^5^ We are reporting what we believe to be the first case of thyroid storm in a patient with COVID-19.

## Case Report

A 25-year-old Hispanic woman with a medical history of obesity (body mass index, 32.0 kg/m^2^), fatty liver disease, gastritis, and obstructive sleep apnea presented to the emergency room in Houston, Texas, in August 2020 with 1 week of intermittent shortness of breath and palpitations. Her symptoms first started over 1 year before presentation, and included palpitations, weight loss, shaking, throat swelling, and dizziness, for which she did not initially seek medical care. She was evaluated at a community health clinic in February 2020 and was told that she had a thyroid problem, but was not started on any medications and did not follow up. During the week leading to this hospital admission, she noticed a significant worsening of her baseline symptoms, including palpitations, diarrhea, anxiety, and heat intolerance, without any known precipitating factor. Review of systems was negative for fever; however, she did have a dry cough and intermittent shortness of breath during this time. While at work, she noticed that her resting heart rate had increased to the 160s, prompting her to come to the hospital.

On physical examination, she was anxious, agitated, and restless with increased fidgeting. Her vitals on admission were as follows: (1) temperature, 98.8 °F; (2) heart rate, 142 beats per minute; (3) blood pressure, 156/92 mmHg; and (4) oxygen saturation, 100% on room air. She had a diffusely enlarged goiter with bruit. Her eyes showed proptosis, chemosis, and lid lag. Extraocular movements were intact, and she had no pain with eye movements. She had a regular cardiac rhythm with tachycardia, and her lungs were clear to auscultation. She had a fine tremor with outstretched arms. There was no pretibial edema. No other abnormalities were noted.

Laboratory results showed a thyroid-stimulating hormone level of <0.01 uIU/L (normal range [NR], 0.45-5.33 uIU/L) and free thyroxine (T4) level of 5.34 ng/dL (NR 0.64-1.42 ng/dL). The total triiodothyronine (T3) level was 654 ng/dL (NR 87-178 ng/dL), and the thyroid-stimulating immunoglobulin level was 7.18 IU/L (NR 0.00-0.55 IU/L). Complete blood count, basic metabolic panel, and aspartate aminotransferase/alanine aminotransferase levels were within normal limits, and the alkaline phosphatase level was elevated at 277 U/L (NR, 34-104 U/L). The total protein and albumin levels were within NRs. HIV and pregnancy testing were negative. She was tested for SARS-CoV-2 via reverse transcriptase–polymerase chain reaction testing, and it resulted as positive for the presence of detectable ribonucleic acid. The initial chest radiograph was normal, and the admission electrocardiogram showed sinus tachycardia with a rate of 164 beats per minute. Thyroid ultrasound revealed heterogeneous echotexture with increased vascularity, highly suggestive of thyroiditis.

The Burch-Wartofsky score was calculated as 55 points: for agitation in the form of restlessness, diarrhea, heart rate of >140 beats per minute, and precipitating event of COVID-19 infection. She was started on 80 mg of propranolol every 4 hours titrated to a heart rate of <90 beats per minute and 200 mg of propylthiouracil every 8 hours. Hydrocortisone (50 mg) every 8 hours was also added to decrease the peripheral conversion of T4 to T3.

**Fig. 1:**
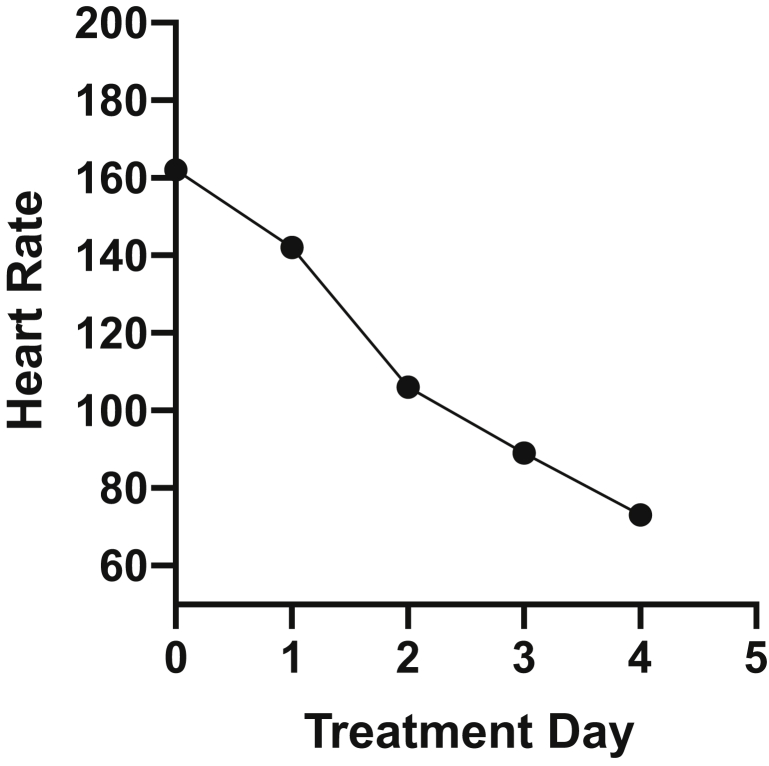
Heart rate during the hospital course.

**Fig. 2:**
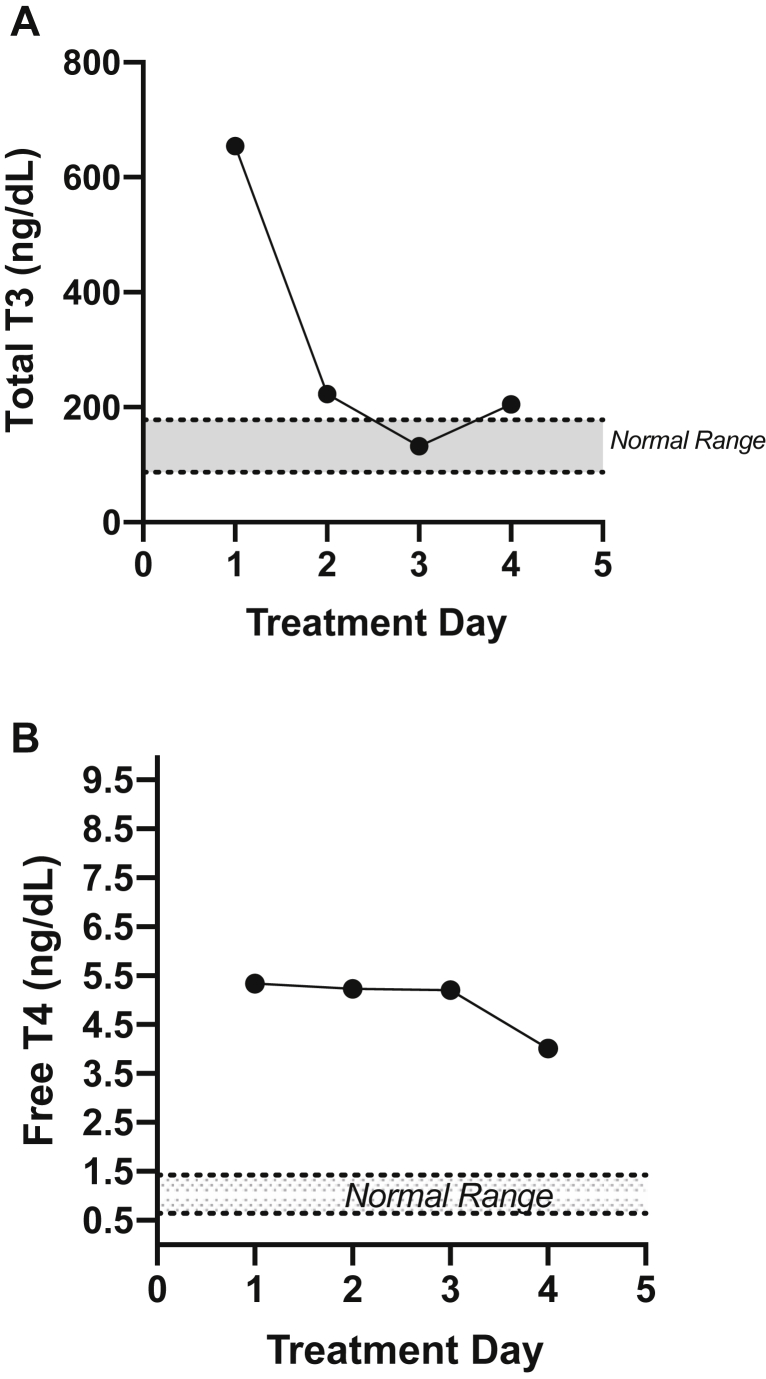
*A,* Total triiodothyronine trend during the hospital course. *B*, Free thyroxine trend during the hospital course.

There was a significant improvement in tachycardia and symptoms after initiation of medications (Fig. 1). The biochemical profile seemed to improve slowly but steadily with a reduction of the total T3 and free T4 levels during hospitalization (Fig. 2 *A* and *B*). Regarding COVID-19, her course was unremarkable. Throughout her hospitalization, she had no decrease in oxygen saturation or radiographic evidence of disease. Her shortness of breath that was present on admission improved with treatment of thyroid storm, but she continued to have a mild dry cough during hospitalization and on discharge. The white blood cell count remained stable and normal throughout admission, despite the diagnosis of COVID-19 and the addition of antithyroid medications. On day 4 of admission, she was discharged home on 20 mg of methimazole 3 times daily, 60 mg of propranolol 2 times daily, and outpatient endocrine follow-up.

## Discussion

Thyroid storms can be a diagnostic challenge as they are defined by largely clinical findings, and there are no universally accepted peer-reviewed criteria for diagnosis.^6^ The point scale by Burch-Wartofsky is most widely used in the United States, but is empirically derived and may overdiagnose some cases of thyroid storm.^7^ In 2012, new diagnostic criteria were proposed by the Japan Thyroid Association, which included the prerequisite of laboratory findings of thyrotoxicosis along with a combination of central nervous system and other organ system dysfunctions.^8^ In the case of our patient, she met the criteria of thyroid storm as defined by both the Burch-Wartofsky point scale and the “TS1” criteria (definite thyroid storm) proposed by the Japan Thyroid Association. However, she notably had no congestive heart failure, pulmonary edema, elevated bilirubin level, or central nervous system manifestation aside from restlessness. Although she met both the technical definitions of thyroid storm, she likely did not represent a life-threatening case at the time of admission.

The precise mechanism of uncomplicated hyperthyroidism or thyrotoxicosis evolving into a thyroid storm is not well known; however, there is almost always a superimposed insult that precipitates it. Increased sensitivity to catecholamines is thought to play a role.^2^ Infection, thyroid and non-thyroid surgeries, parturition, major trauma, and the use of certain drugs are all known causes. Although autoimmune thyroid disease in itself is not linked to an increased risk of COVID-19, uncontrolled thyrotoxicosis with SARS-CoV-2 infection could precipitate a more severe course of thyroid disease. In our patient, it is possible that infection with SARS-CoV-2 pushed her from stable thyrotoxicosis to thyroid storm. A July 2020 retrospective study in the *European Journal of Endocrinology* suggested that COVID-19 may be associated with a high risk of thyrotoxicosis due to the relationship with immune system activation induced by SARS-CoV-2 infection.^9^ It was recently reported that patients with COVID-19 show high levels of pro-inflammatory cytokines and chemokines.^10^^,^^11^ The levels of pro-inflammatory cytokines such as IL-6 and IL-8 have been shown to be elevated in untreated thyrotoxicosis and fall as thyroid levels normalize with medical treatment.^12^

In our patient, we used the Burch-Wartofsky scoring system along with clinical and biochemical presentations to recognize the thyroid storm.^13^ The presence of a precipitating event (SARS-CoV-2 infection, albeit a mild case) bumped her score from borderline thyroid storm to overt thyroid storm. Although she was previously told she had a thyroid problem, she had not been started on any antithyroid therapy, so she had no alternative precipitating factor such as medication noncompliance. Regarding the positive COVID-19 test, we believe that her test can be interpreted as a true positive. According to the *BMJ*, the probability of actually having COVID-19 with a positive reverse transcriptase–polymerase chain reaction test is 98%.^14^ Additionally, she had exposure risk as she worked in a health care clinic, and was symptomatic with dry cough and shortness of breath.

Early recognition of thyroid storm, in part due to prompt COVID-19 testing, helped to initiate therapy that is essential in reducing mortality. Treatment in our patient was guided toward inhibiting thyroid hormone synthesis, counteracting the peripheral effects of thyroid hormones, and reducing systemic effects including tachycardia. As infections due to SARS-CoV-2 continue to be prevalent, it is essential to test all symptomatic patients for COVID-19, as it may guide clinical decision-making in situations such as thyroid storms. Although there have been case reports of COVID-19-related subacute thyroiditis, this appears to be the first documented case of thyroid storm as defined by the Burch-Wartofsky criteria in a patient positive for COVID-19.^5^ As we continue to learn more about the novel coronavirus, a more definitive link regarding its role in thyrotoxicosis may emerge.

## Disclosure

The authors have no multiplicity of interest to disclose.
